# Decoding the chromatin proteome of a single genomic locus by DNA sequencing

**DOI:** 10.1371/journal.pbio.2005542

**Published:** 2018-07-13

**Authors:** Tessy Korthout, Deepani W. Poramba-Liyanage, Ila van Kruijsbergen, Kitty F. Verzijlbergen, Frank P. A. van Gemert, Tibor van Welsem, Fred van Leeuwen

**Affiliations:** Division of Gene Regulation, Netherlands Cancer Institute, Amsterdam, The Netherlands; National Cancer Institute, United States of America

## Abstract

Transcription, replication, and repair involve interactions of specific genomic loci with many different proteins. How these interactions are orchestrated at any given location and under changing cellular conditions is largely unknown because systematically measuring protein–DNA interactions at a specific locus in the genome is challenging. To address this problem, we developed Epi-Decoder, a Tag-chromatin immunoprecipitation-Barcode-Sequencing (TAG-ChIP-Barcode-Seq) technology in budding yeast. Epi-Decoder is orthogonal to proteomics approaches because it does not rely on mass spectrometry (MS) but instead takes advantage of DNA sequencing. Analysis of the proteome of a transcribed locus proximal to an origin of replication revealed more than 400 interacting proteins. Moreover, replication stress induced changes in local chromatin proteome composition prior to local origin firing, affecting replication proteins as well as transcription proteins. Finally, we show that native genomic loci can be decoded by efficient construction of barcode libraries assisted by clustered regularly interspaced short palindromic repeats and CRISPR-associated protein 9 (CRISPR/Cas9). Thus, Epi-Decoder is an effective strategy to identify and quantify in an unbiased and systematic manner the proteome of an individual genomic locus by DNA sequencing.

## Author summary

DNA is packaged by proteins into a structure called chromatin. This packaging plays an important role in gene transcription, DNA replication, and DNA repair. The regulation of these processes is determined by the interplay of proteins that physically interact with specific loci. Although many chromatin proteins have been identified, it remains a challenge to comprehensively identify all the chromatin interactions at any specific locus by protein-based methods such as capture mass spectrometry (MS). Here, we take advantage of DNA barcoding technologies and high-throughput sequencing to tackle this problem. We developed Epi-Decoder, a method that identifies chromatin–protein interaction at a single-copy locus in budding yeast. Epi-Decoder was successfully applied at 3 different genomic loci, resulting in comprehensive overviews of local chromatin interactomes. Most factors identified are known to be involved in chromatin-associated processes such as transcription and DNA replication. We also identified unexpected chromatin–protein interactions. Furthermore, capturing the dynamic chromatin interactome by changing physiological conditions provided insight into the local response to stress. In conclusion, Epi-Decoder is an effective tool for measuring the local proteome of a specific genomic locus by DNA sequencing.

## Introduction

The chromatin at any given location in the genome is a dynamic entity that likely involves the interaction of many different proteins and nonprotein factors. The essential and complex processes of transcription, replication, and repair require major chromatin rearrangements to access and use the genome [1,2]. Furthermore, the genome’s chromatin is under the influence of signals that can relay information of cellular events or states to the genome and vice versa [3,4]. Fully understanding the chromatin regulatory mechanisms in the cell will require comprehensive knowledge of the full set of proteins that bind at individual genomic loci. Although many chromatin factors have already been identified by genetics and protein–protein interaction studies, direct, unbiased, and comprehensive analyses of chromatin interactions at specific genomic loci has remained a major challenge [5,6]. A commonly considered strategy towards solving this problem is introducing an affinity handle at a locus of interest, purifying the locus (capture), and analysing the copurified proteins by mass spectrometry (MS) [7,8]. However, a major challenge with capture MS is the need for very high levels of enrichment of the locus of interest versus the rest of the large genome while at the same time obtaining sufficient amounts of material for comprehensive and quantitative MS analysis [5,6]. For example, in a model organism with a small genome such as yeast, purification of a 1 kb locus with its associated proteins from an entire genome of approximately 30 Mb requires a 30,000-fold purification.

Here, we present an independent approach, Epi-Decoder, with which the interactome of a single-copy locus is determined by DNA sequencing instead of MS. In this approach developed in yeast, a library of clones is generated in which each clone harbours at least one DNA barcode at a fixed locus and one protein tagged at its endogenous locus with a common epitope tag. Following chromatin immunoprecipitation (ChIP) for the common tag on a pool of cells, the barcodes of the coimmunoprecipitated DNA are counted by high-throughput sequencing, and the amount of barcode recovered serves as a read-out for the amount of the corresponding specific protein binding at the barcoded locus. In contrast with proteins, DNA barcodes can be amplified prior to counting, boosting the sensitivity of detection.

Epi-Decoder of a single transcribed locus in yeast identified more than 400 chromatin-interacting proteins, enabled the identification of differences in protein binding between the 5’ and 3’ end of a gene, and demonstrated a chromatin rewiring in response to physiological changes. We also demonstrate that Epi-Decoder can be applied to a locus of interest by harnessing clustered regularly interspaced short palindromic repeats and CRISPR-associated protein 9 (CRISPR/Cas9)-mediated genome engineering. Thus, Epi-Decoder provides an efficient method to provide a comprehensive map of the dynamic proteome of a single-copy locus. Moreover, it is an orthogonal approach to capture MS because acquisition of quantitative and qualitative information on protein binding does not involve MS but is obtained by DNA sequencing.

## Results

### Epi-Decoder: Measuring protein binding by DNA barcode counting

**Fig 1:**
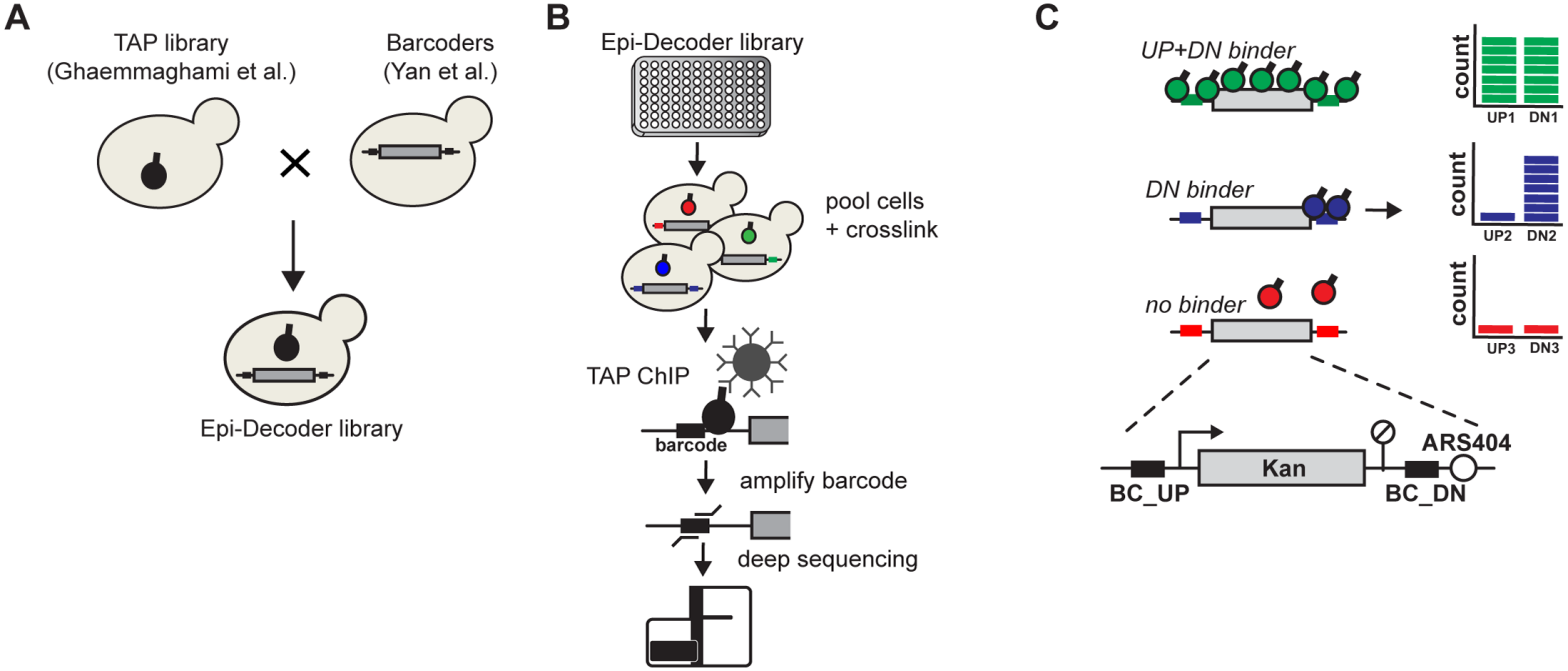
Outline of Epi-Decoder. (A) Construction of the Epi-Decoder library: a library of strains encoding TAP-tagged proteins was crossed to a Barcoder library by using standard SGA procedures. (B) Experimental set-up of Epi-Decoder: colonies of an arrayed library are pooled in a flask, expanded, subjected to crosslinking, and used for a TAP-ChIP. The coimmunoprecipitated barcodes are amplified and counted by massive parallel sequencing. Barcodes of input DNA are counted to control for the presence of each barcode in the pool. Indexed input and ChIP samples of multiple pools can be combined into 1 master pool before sequencing. (C) Protein binding is measured by barcode counting. The Barcoder locus consists of a *KanMX* resistance marker transcribed by the *AgTEF1* promoter flanked by 2 unique barcodes and replacing the HO gene. The BC_UP is promoter-proximal and the BC_DN terminator-proximal and close to (50 bp) an origin of replication (ARS404). BC_DN, downstream barcode; BC_UP, upstream barcode; ChIP, chromatin immunoprecipitation; SGA, synthetic genetic array; TAP, Tandem Affinity Purification.

To develop a strategy for systematic and comprehensive decoding of the interactome of a single genomic locus, we asked whether DNA barcode technology can be harnessed to solve this challenging proteomics problem. We and others previously showed that short DNA barcodes (≤20 bp) integrated in the genome and embedded in chromatin can serve as molecular identifiers of the chromatin state they are in [9–11]. Building on that notion, we used synthetic genetic array (SGA) technologies [12,13] to create arrayed yeast libraries that allow for high-throughput and direct assessment of chromatin states of many cell clones in parallel. In these libraries, each clone contains a pair of known unique barcodes flanking a constitutively transcribed kanamycin resistance gene (*KanMX*) under control of the Ashbya gossypii (Ag) *TEF1*-promoter and -terminator at the *HO* locus [14] as well as a known Tandem Affinity Purification (TAP)-tagged protein expressed from its endogenous locus [15] (Fig 1A). The Epi-Decoder libraries used here cover approximately 4,000 TAP-tagged proteins (S1 Table) of the approximately 5,600 proteins encoded in the yeast genome [16]. Following pooling of the barcoded TAP-tagged strains, cells are incubated with formaldehyde to crosslink proteins to DNA and subjected to ChIP (Fig 1B). From the coimmunoprecipitated DNA and the input samples, the barcoded regions are amplified (S1 Fig), and the barcodes are identified and quantified by parallel sequencing (Fig 1B). In this set-up, which we call Epi-Decoder, the abundance of a barcode (ChIP/input) reports on the crosslinking of the corresponding TAP-tagged protein to its own barcoded region for every TAP-tagged clone in the pool (Fig 1C). The reporter gene at the *HO* locus is flanked by 2 barcodes that can be analysed in parallel but that have different chromatin contexts: the upstream barcode (BC_UP) is located in the promoter region, whereas the downstream barcode (BC_DN) lies in the terminator region and in close proximity to an origin of replication (Fig 1C). Thus, Epi-Decoder analysis of the barcoded *KanMX* gene at the HO locus results in an inferred binding score for the vast majority of the proteins present in the yeast genome at 2 proximal but distinct genomic loci.

### Epi-Decoder identifies many chromatin-associated factors

**Fig 2:**
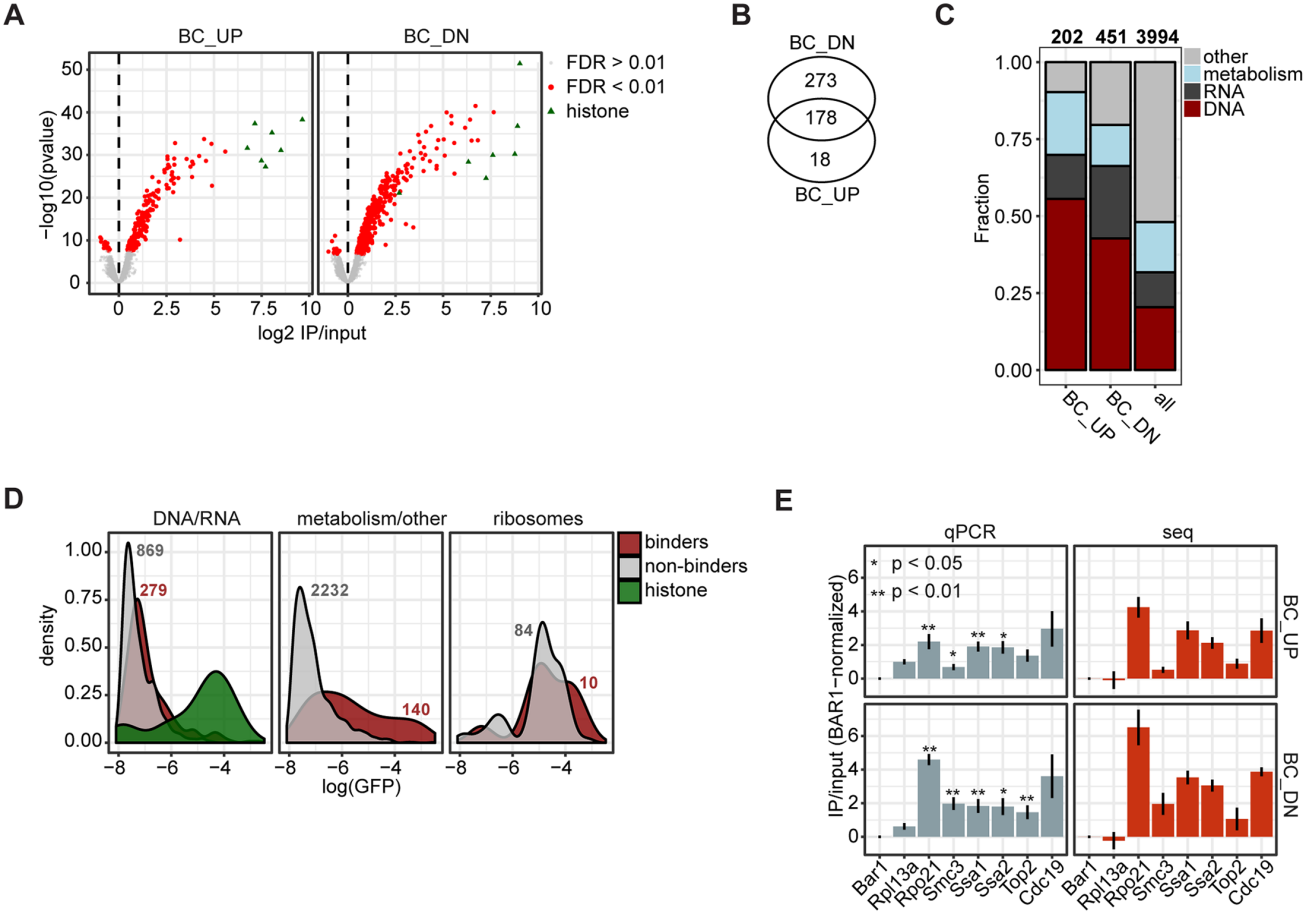
Identifying chromatin binders with DNA barcode counting. (A) Volcano plots showing the average log2 ChIP/input versus significance (*p*-value) calculated for 3,994 factors for BC_UP and 3,995 factors for BC_DN across 6 independent biological experiments. The red colour indicates factors with a Benjamini-Hochberg–adjusted *p*-value < 0.01. Green triangles represent the barcodes associated with the TAP-tagged histone proteins in the library. (B) Venn diagram indicating the number of factors that are significant (FC > 0; FDR < 0.01) at BC_UP, BC_DN, or both. (C) Bar plot showing the fraction of factors in 4 different categories that were determined by using GO annotations (see Methods). The first 2 bars show the fractions in the group of binders (at either BC_UP or BC_DN). The right bar shows the fraction in the background set (the entire library). (D) Density plot of GFP abundance using data from the Cyclops database (http://cyclops.ccbr.utoronto.ca/). The binders were defined as before (FC > 0; FDR < 0.01), and the categories are similar to those in panel C. The ribosome category was defined based on the CYC2008 database (http://wodaklab.org/cyc2008/). Histones are shown separately; they bind at the DNA and are highly abundant. The numbers indicate the size of each group. (E) ChIP-qPCR of selected TAP-tagged strains from the barcoded KanMX Epi-Decoder library, with specific primers in proximity to BC_UP and BC_DN. To compare the barcode counts with ChIP-qPCR signal, the samples were normalized to the Bar1-TAP signal (Bar1 is not expressed in these cells) before calculating ChIP/input. The average of 3 biological replicates is shown; the error bars indicate the SD. Rpl31A—a highly expressed ribosomal subunit frequently used in anchor away studies to deplete proteins from the nucleus—was used as a negative control and as a reference to determine binding specificity in the qPCR analysis. Rpo21 is the largest subunit of RNA polymerase II, Smc3 is a cohesin subunit, Ssa1 and Ssa2 are HSP70 chaperones, Top2 is topoisomerase II, and Cdc19/Pyk1 is pyruvate kinase. The *p*-values from a two-sided *t* test compared to Rpl13A are indicated by asterisks. Underlying data for Fig 2C and Fig 2E in S1 Data. BC_DN, downstream barcode; BC_UP, upstream barcode; ChIP, chromatin immunoprecipitation; FC, fold change; FDR, false discovery rate; GFP, green fluorescent protein; GO, gene ontology; IP, immunoprecipitation; qPCR, quantitative PCR; TAP, Tandem Affinity Purification.

For BC_UP and BC_DN, we identified the immunoprecipitated barcodes with significantly different counts compared to background (red dots in Fig 2A), based on 6 biological replicates (S2A Fig). The vast majority of significantly different barcodes showed a positive binding score (Fig 2A, red dots, ChIP/input > 0). Here, we refer to the factors associated with those barcodes as binders. Together, we identified 469 binders of which 18 were specific for BC_UP and 273 for BC_DN (Fig 2B, S1 Table). Significantly depleted factors (Fig 2A, red dots, ChIP/Input < 0) were not expected because proteins cannot bind less to their barcodes than negative controls such as nonexpressed or nontagged proteins. The low number of significantly depleted factors indicates a low rate of false discovery, in agreement with our stringent cut-off (false discovery rate [FDR] < 0.01). Among the binders, the 4 canonical histone proteins (represented by 7 histone genes in the library) were among the most enriched proteins at both barcodes. Furthermore, 267 out of the 469 binders had gene ontology (GO) terms related to DNA binding (Fig 2C, S2B Fig), confirming that Epi-Decoder provides an effective approach for identifying chromatin-interacting factors. In addition to known DNA-interacting proteins, we found a substantial number of factors involved in RNA processing and cellular metabolism. RNA processing events such as capping, splicing, cleavage, and polyadenylation are all processes known to occur in close conjunction with transcription and hence in proximity to DNA [17], providing an explanation for the recovery of barcodes associated with RNA processing proteins. The presence of factors involved in cellular metabolism cannot be explained by general associations with transcription, although recent studies have suggested various roles of metabolic enzymes in the nucleus [4,18,19] (and see Discussion). Given the fact that some of these factors are known to be highly expressed, we investigated the possibility that their chromatin interactions were determined by protein abundance. Overall, chromatin binders were generally more highly expressed than nonbinders (Fig 2D). However, high protein expression level alone was not sufficient for binding—many chromatin binders were not highly abundant, and many highly abundant proteins were nonbinders. For example, ribosomal proteins are highly abundant, but most of them were not detected as chromatin interactors even though ribosomal proteins traffic through the nucleus to form ribonucleoprotein complexes for ribosome assembly.

Furthermore, for a selected panel of factors reflecting different classes of binders and different expression levels, we could quantitatively confirm the positive and negative Epi-Decoder results by ChIP-quantitative PCR (qPCR) and immunoblot analysis of individual clones (Fig 2E, S2C and S2D Fig). Finally, we excluded the possibility that leaking of cytosolic factors into the nucleus due to the presence of methanol in formaldehyde solutions was a cause of the crosslinking of metabolic enzymes to chromatin. Using formaldehyde with or without methanol as a stabilizer resulted in nearly identical binding profiles (S2E Fig). Whether or not the binding of metabolic enzymes to chromatin is biologically significant will require further in-depth studies and functional experiments. Together, our results suggest that the barcode counts obtained by Epi-Decoder accurately and quantitatively report on the efficiency of protein crosslinking at the barcoded loci and capture histones and other core chromatin proteins, as well as additional factors. We note that in the Epi-Decoder set-up, proximity to DNA, genomic distance from the barcode, protein abundance, and cell-to-cell variation are among the factors that can influence the measured quantitative binding scores.

### Different interactomes of the promoter and terminator regions

**Fig 3:**
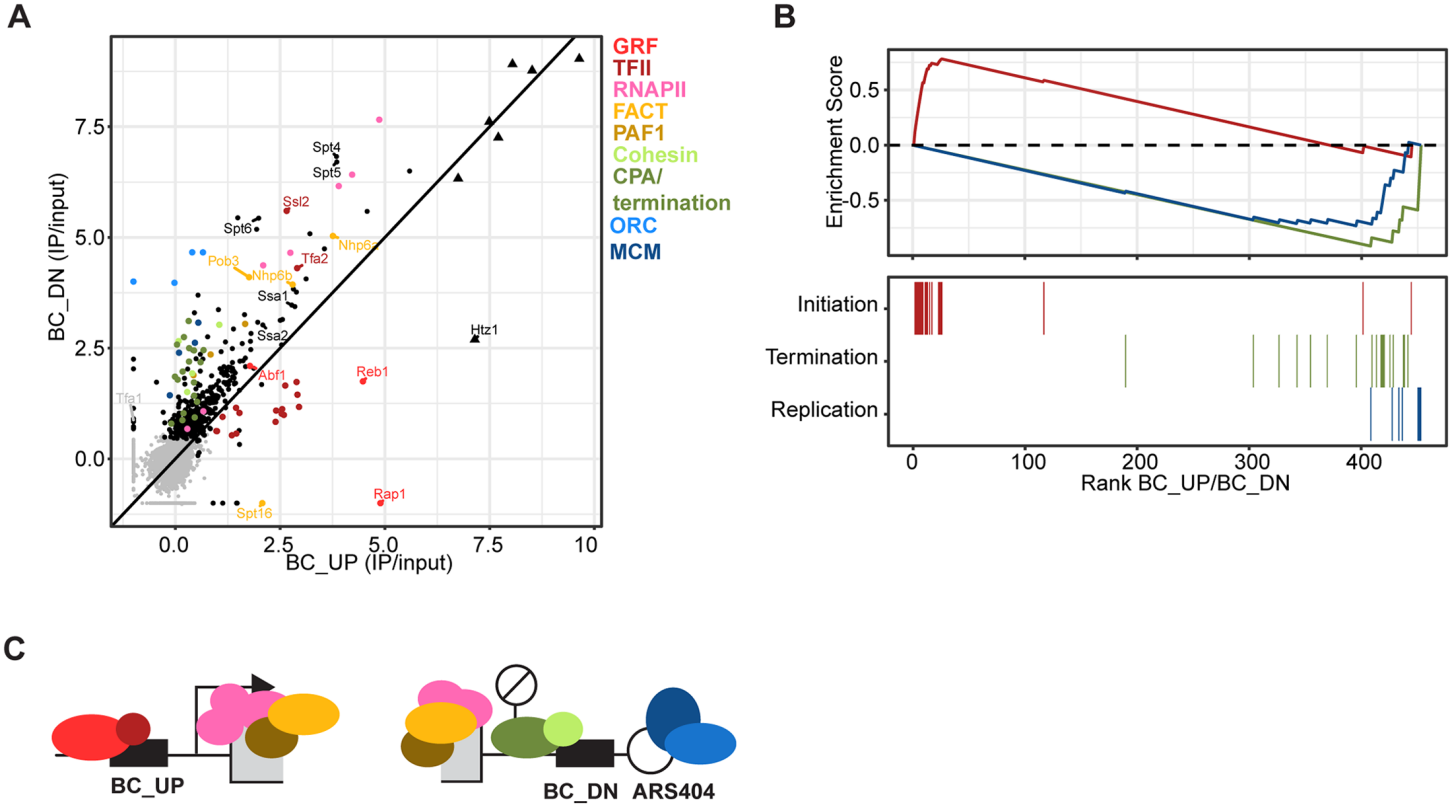
Capturing the chromatin interactome with Epi-Decoder. (A) Scatter plot of ChIP/input values, which can be interpreted as binding scores, of BC_UP versus BC_DN. For some factors, only the BC_UP or BC_DN value was available. In these cases, the missing value was set to −1 in order to still visualize the remaining value. Factors are coloured based on known functions or complexes. Triangles represent histone proteins. The average values of 6 replicates are shown. (B) Enrichment plot for 3 categories: initiation (TFIID-E and GRFs), termination (Cleavage and Polyadenylation and THO complex), and replication (ORC and MCM) factors. Binders were ranked based on the BC_UP/BC_DN ratio. The top part shows the running sum enrichment for each category. Initiation factors were significantly enriched at BC_UP (*p* = 3.18^−3^) and termination and replication at BC_DN (*p* = 2.03^−7^ and *p* = 1.06^−5^). The bottom lines indicate the factors represented in the categories. (C) Illustration of the different protein complexes that Epi-Decoder identified at the barcoded *KanMX* gene at the *HO* locus. BC_DN, downstream barcode; BC_UP, upstream barcode; ChIP, chromatin immunoprecipitation; GRF, general regulatory factor; IP, immunoprecipitation; MCM, minichromosome maintenance; ORC, origin recognition complex.

To determine whether Epi-Decoder can be used to identify locus-specific proteins, we next compared the significantly enriched factors for BC_UP to those of BC_DN. For this purpose, we plotted the binding scores for BC_UP versus BC_DN and color-coded enriched factors according to the main chromatin functions or complexes expected at this locus (Fig 3A). We observed that proteins within the same complex or process tend to cluster together. This strongly suggests that Epi-Decoder does not just detect binding events but also provides quantitative information about protein occupancy.

Several factors and complexes were shared between the 2 locations. Besides histones, strongly enriched factors at both barcodes include subunits of RNA polymerase II and several other factors and complexes with a well-known role in transcription elongation such as DSIF, Elf1, FACT, Spt6, and the PAF-C complex (Fig 3A) [20,21]. However, BC_UP and BC_DN also showed substantial quantitative interactome differences reflecting their different functional states and suggesting that many proteins show locus-specific binding behaviour (Fig 3B). At BC_UP at the 5’ end of the gene, histone variant H2A.Z (Htz1) was strongly enriched, which is in agreement with the enrichment of H2A.Z in promoter regions [22], which we confirmed for this locus using an antibody that recognizes the endogenous H2A.Z protein (S3A Fig). In addition, BC_UP showed enrichment for general regulatory factors Reb1 and Rap1. This is in agreement with the known Rap1 binding site in the *TEF1* promoter of the *KanMX* gene [23] and the role of Reb1 as a general chromatin organizer of regulatory regions [20]. BC_UP also showed enrichment for the basal transcription factors TFIID, -E, -F, and -H, which form the pre-initiation complex together with RNA polymerase II [21,24].

At the 3’ end, BC_DN showed enrichment for factors involved in transcription termination and mRNA cleavage and polyadenylation, an important step in mRNA 3’ end formation [25]. The Cohesin complex was also enriched at BC_DN. Finally, we observed strong binding of the evolutionarily conserved origin recognition complex (ORC) and minichromosome maintenance (MCM) complexes at BC_DN, which is in agreement with the proximal origin of replication sequence at which ORC and then MCM are loaded to assemble the prereplication complex [26,27]. BC_DN-specific binding of Orc1 was confirmed by ChIP-qPCR (S2C Fig). Thus, in addition to common binders, Epi-Decoder revealed locus-specific enrichment of factors (Fig 3C). This confirms that, while the barcodes are in the same genomic region, they are in distinct functional contexts that can be separated by this approach even though they are only 1.5 kb apart. This level of resolution was robust because the BC_UP- and BC_DN-specific binding pattern was maintained when the average chromatin fragment size was increased or decreased by applying shorter and longer shearing time, respectively (S3B and S3C Fig).

Not all factors followed the expected distribution (Fig 3A). Tfa2, the small subunit of the heterodimeric basal transcription factor TFIIE, and Ssl2, the double-stranded DNA (dsDNA) translocase subunit of the basal transcription factor TFIIH, were found at BC_UP but were also strongly enriched at BC_DN. 3’ binding of Tfa2 and Ssl2 has been observed at other genes as well [24,28], but the significance remained uncertain. Because in Epi-Decoder all proteins have the same tag and are assessed simultaneously in a pool in a quantitative manner, the deviant binding pattern observed here cannot be explained by antibody issues or experimental and strain differences and therefore strongly suggests that Tfa2 and Ssl2 have special roles or positions within their complex or have noncanonical functions outside their complex.

### Conditional local proteomes: Chromatin rewiring in hydroxyurea

The chromatin interactome defined by Epi-Decoder confirmed that the barcodes flank an actively transcribed gene in close proximity to and transcribing towards an origin of replication that is licensed with the prereplicative complex. This conformation is of special interest because, upon firing of the origin, replication fork progression will require negotiation with the transcription machinery, potentially leading to collisions [29–33]. It has been proposed that, in order to avoid collisions, RNA polymerase II is degraded at a subset of genes that is about to be replicated [34]. Because this recently described process of chromatin adaptation is still poorly understood, we used Epi-Decoder to determine, in a comprehensive and unbiased manner, how the barcoded HO locus interactome changes when cells are arrested in early S phase in hydroxyurea (HU) (S4A Fig), the condition in which RNA polymerase II degradation has been observed [34]. HU affects replication-fork progression by reducing the supply of deoxyribonucleotide triphosphates (dNTPs) and by the generation of reactive oxygen species [35].

**Fig 4:**
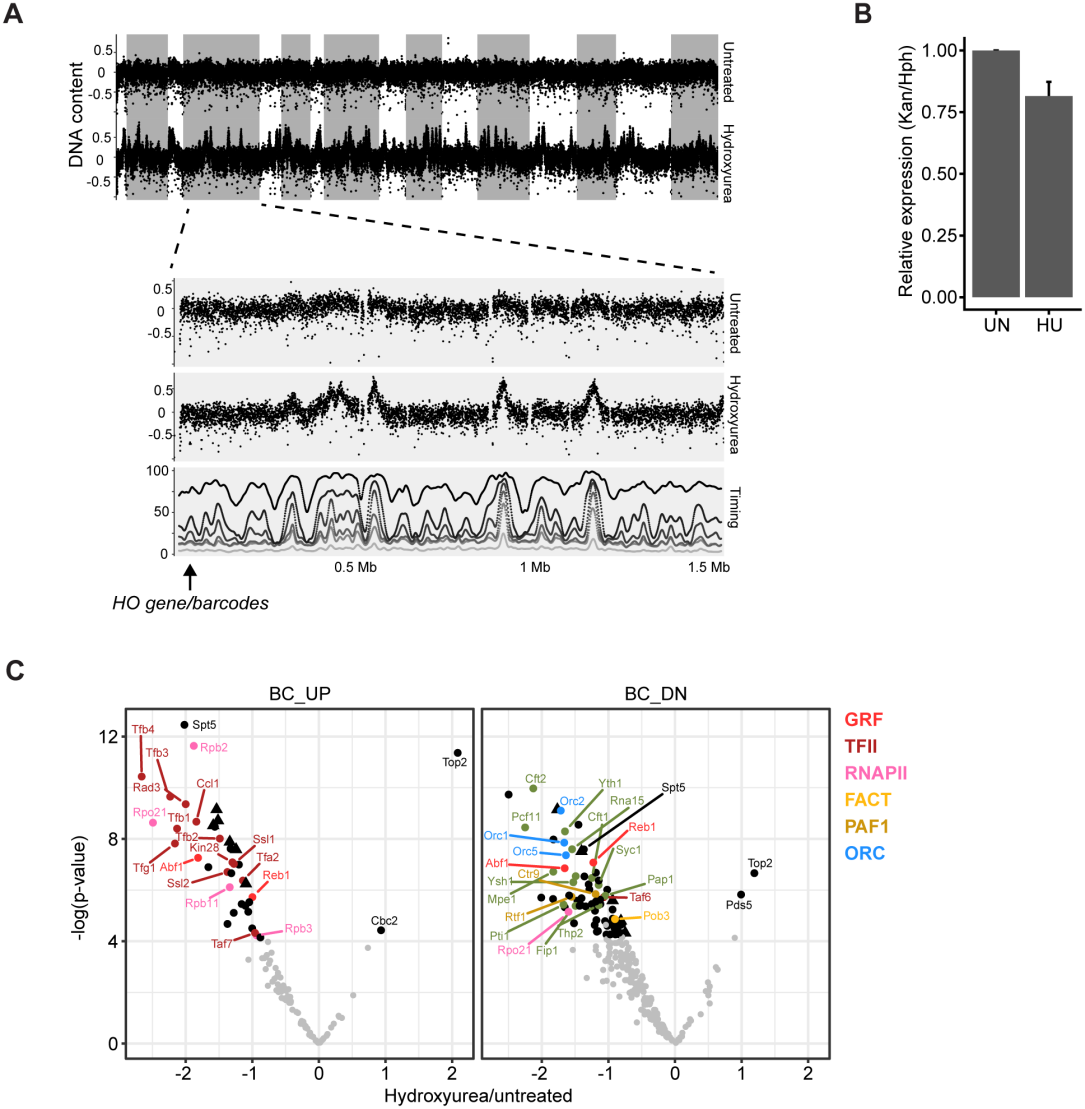
Chromatin rewiring upon HU treatment. (A) DNA regions replicated in the HU arrest were determined by DNA copy number assessment. The DNA coverage in bins of 200 bp is plotted across the genome for untreated and HU-treated samples. The lower panel shows a zoom-in of chromosome IV, which contains the barcoded locus on the left arm (indicated by the arrow). Replication timing in the absence of HU was obtained from Alvino and colleagues [36]. The percentage of replicated DNA is plotted for each bin at 10, 12.5, 15, 17.5, 25, and 40 minutes after release from a G1 arrest, as indicated by the shades of grey. (B) RT-qPCR shows relative mRNA expression of *KanMX/HphMX* in untreated and HU-treated samples. The untreated samples were set to 1. The average of 3 biological replicates is shown; error bars indicate SD. (C) Volcano plots showing the ratio of binding in HU-treated/untreated for factors that were significantly enriched in either one or both of the conditions. The coloured dots are factors with significantly different binding scores (FDR < 0.05). BC_DN, downstream barcode; BC_UP, upstream barcode; FDR, false discovery rate; HU, hydroxyurea; ORC, origin recognition complex; RT-qPCR, quantitative reverse transcription PCR.

We first confirmed previous observations [36] that, in HU, early origins have fired but that the middle to late barcoded HO origin (ARS404) has not fired yet (Fig 4A). Despite the absence of initiation of replication, Epi-Decoder revealed multiple changes in the chromatin proteome; 40 proteins showed a significantly different binding score in HU at BC_UP, and 79 were significantly different at BC_DN—their altered abundance could not be explained by changes in protein levels (S4B Fig). Firstly, we observed lower mRNA levels of the barcoded *KanMX* gene compared to the *HphMX* gene, which expresses a different mRNA (Hph instead of Kan) from the same *AgTEF1* promoter but at a different genomic location (*HphMX* replaces the *CAN1* gene at chromosome V) and not in proximity to an origin of replication (Fig 4B). This was accompanied by a reduction in occupancy of multiple RNA polymerase II subunits at the barcoded gene, extending the previous findings that RNA polymerase II is degraded at a subset of genes that has been or is about to be replicated (Fig 4C). Secondly, the chromatin response was not restricted to RNA polymerase II because other general transcription proteins involved in capping, initiation, elongation, and termination were also reduced, showing that the reduction of transcription involves a broad range of changes in the (co)transcriptional machinery (Fig 4C). Thirdly, we observed additional changes that were not directly related to transcription but indicate topological alterations (Fig 4C and S4C Fig). Topoisomerase II (Top2) binding was strongly increased at both ends of the *KanMX* gene in HU. Top2 is the main enzyme releasing topological stress during S phase [37] and is targeted to nucleosome-free DNA during replication stress [38]. Pds5, the cohesin maintenance factor was also increased at BC_DN, and a closer inspection of the cohesin complex indicates that 3 of the 4 cohesin subunits in the library showed moderately increased occupancy as well, suggesting stabilisation of cohesin binding in this region (S2 Table). Finally, all the subunits of ORC in the library showed decreased binding at BC_DN, while occupancy of MCM was unaltered. ORC proteins have been suggested to remain bound to origins throughout the yeast cell cycle [39,40], possibly being negatively influenced by MCM proteins in G1 [41]. The direct and quantitative comparison of all origin-proximal factors by Epi-Decoder suggests that the interaction of ORC proteins with the origin is compromised in S phase prior to firing but that this cannot be explained by increased MCM–protein occupancy. Therefore, our results show that ORC subunits interact more dynamically with chromatin throughout the yeast cell cycle than expected but are in agreement with the behaviour of ORC in other organisms [39,42]. How lower ORC binding influences origin firing and subsequent fork progression of this locus remains unknown. Recent in vitro reconstitution experiments demonstrated that the MCM complex is stably bound to DNA once assembled and also competent for replication, even after removal of ORC proteins, suggesting that origin firing per se may not be affected [43].

In summary, Epi-Decoder uncovered condition-dependent composition of a local chromatin proteome. At a transcribed gene next to a licensed origin, arrest in HU led to tuning down of transcription by a general reduction of transcription protein binding, which is accompanied by increased occupancy of topology factors and altered stoichiometry of replication proteins.

### Application of Epi-Decoder to a native locus

**Fig 5:**
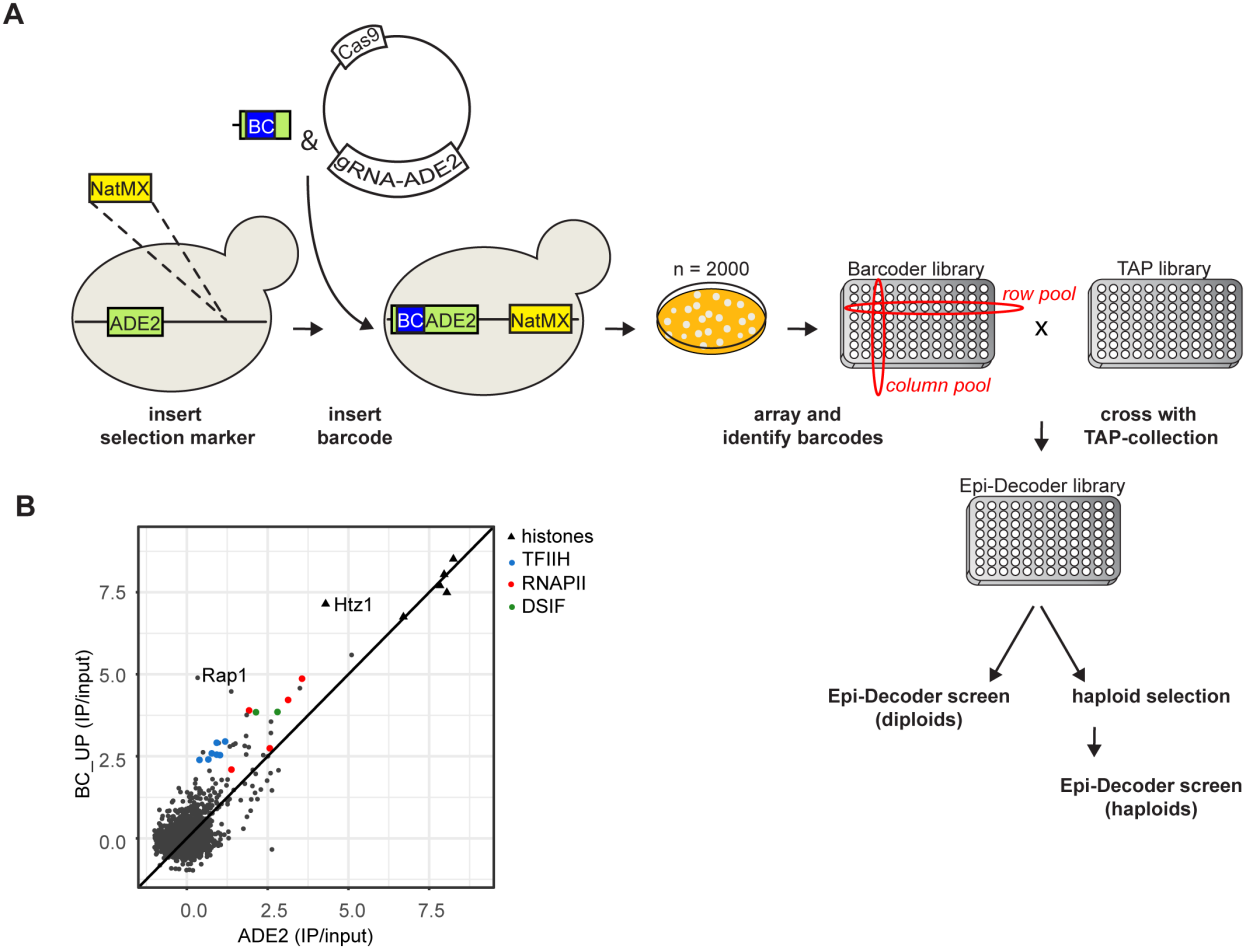
Application of Epi-Decoder to a native genomic locus. (A) Procedure to generate a custom Epi-Decoder collection. A selection marker (*NatMX*) was inserted in proximity to the locus of interest, downstream of the to-be-barcoded *ADE2* gene. Subsequently, a Cas9- and gRNA-expressing vector was introduced in this strain together with a repair template that is composed of a 15 bp random barcode-sequence (BC) flanked by 40 bp arms with homology to the gRNA target site, here at the 5’ end of the *ADE2* coding sequence (details see S5A Fig). Transformants, the vast majority of which represent barcoded clones, were transferred to an arrayed format. Rows and columns were pooled for identifying barcodes and their location on the array by high-throughput sequencing. The barcoder library was mated and crossed with the TAP-tagged protein library (see Fig 1) to generate heterozygous diploids and then haploids, after which Epi-Decoder screens could be performed. For diploid screens, the insertion of a selectable marker genetically linked to the barcode can be omitted. (B) Epi-Decoder results of BC_5’-ADE2 (*n* = 2, average) in diploid cells were compared to BC_UP in haploid cells (Fig 2). Binding scores were visualized in a scatterplot with histones shown as triangles and classes of proteins coloured: TFIIH, RNAPII, DSIF. BC, barcode; BC_5’-ADE2, barcode in 5’ region of *ADE2*; BC_UP, upstream barcode at HO locus; Cas9, CRISPR-associated protein 9; gRNA, guide RNA; IP, immunoprecipitation; TAP, Tandem Affinity Purification.

We showed that Epi-Decoder effectively measures DNA–protein interactions at a barcoded reporter gene. Next, we aimed to establish versatility, i.e., the ability to apply Epi-Decoder to any native genomic locus of interest. To this end, we set up a protocol that facilitates the generation of new barcoded libraries by implementation of CRISPR/Cas9 (Fig 5A). In *Saccharomyces cerevisiae*, a double-strand break induced by targeted Cas9 is generally lethal unless it can be repaired by homologous recombination [44–47]. Therefore, we expected that transforming a strain with Cas9, guide RNA (gRNA), and a barcode-containing repair template would result in efficient barcode incorporation (Fig 5A). For a proof of concept, we chose to insert a 15-bp barcode in the transcribed region of the *ADE2* gene (BC_5’-ADE2), 57 bp downstream of the start of the *ADE2* coding sequence. Because disruption of *ADE2* causes the accumulation of a red pigment in the cell, it provided a direct visual read-out to confirm the efficiency of the barcode insertion [44,48]. Random colonies were selected and transferred to an arrayed format to enable crossing to the TAP-tagged library and, in parallel, to identify the integrated barcode at each location. The vast majority of the colonies turned red and contained a barcode insertion, confirming that CRISPR/Cas9 can provide a highly efficient barcoding strategy. The new BC_5’-ADE2 collection contained 1,974 strains with a unique barcode at the 5`end of *ADE2*. It was crossed with the TAP-tagged protein collection, which resulted in 3,604 barcode-TAP-tagged protein combinations. A *NatMX* cassette integrated downstream of the *ADE2* gene was used to select for the barcoded locus during the genetic crosses without interfering with the chromatin context of the barcode.

Here, the Epi-Decoder screen for BC_5’-ADE2 was performed on diploid cells, in which the TAP-tagged allele and the barcoded allele are both present in a heterozygous fashion. As expected, histones and other general chromatin factors were detected (Fig 5B and S5 Table). Furthermore, we detected at BC_5’-ADE2 proteins associated with active transcription such as RNAPII subunits, transcription initiation factors, and transcription elongation factors, consistent with the promoter-proximal location of the barcode. Indeed, the binding pattern of BC_5’-ADE2 was very similar to that of BC_UP, which is also in a promoter-proximal location (Fig 5B). Together, this confirmed the successful application of Epi-Decoder to a new, native barcoded locus. BC_UP and BC_5’-ADE2 also showed some differences. Rap1, which is a strong binder at the Rap1-controlled *AgTEF1* promoter [23], was not detected at the 5’ end of the *ADE2* gene, which is in agreement with what is known about Rap1 binding across the genome [49]. Furthermore, transcription-initiation and -elongation proteins were generally recovered with lower scores at BC_5’-ADE2 than at BC_UP, which is in agreement with the lower transcript levels found for *ADE2* versus *TEF1* and *AgTEF1* promoter-controlled transcripts (S5B Fig and [50]). Thus, by taking advantage of efficient CRISPR/Cas9-mediated barcode library construction, Epi-Decoder can uncover the local chromatin proteomes of native loci of interest.

## Discussion

Strategies aimed at identifying proteins bound at specific genomic loci frequently involve affinity capture combined with MS [5,6]. Affinity handles generally involve engineering the locus by introducing binding sites for a tagged protein, capturing a native locus by using oligo-capture, or targeting inactive Cas9-fusion proteins. Important progress has been made for multicopy DNA loci such as repetitive DNA elements [7,8]. Recently, oligo-capture [51] and CRISPR/Cas9 technology [52,53] have been applied to identify proteins at single-copy genomic loci. However, the problem of assessing a unique locus in a comprehensive and quantitative manner by MS has not been solved, and many challenges remain, a major one being the high degree of purification of the locus of interest while obtaining sufficient material for good coverage in MS analysis [6]. Epi-Decoder provides a powerful and orthogonal strategy in which the very challenging proteomics problem of decoding the chromatin proteome of a single genomic locus is addressed by DNA sequencing. We demonstrate that Epi-Decoder enables an unbiased, comprehensive, and quantitative analysis of protein occupancy of a single locus in budding yeast.

In addition to known and expected core chromatin proteins, we observed significant binding scores for many proteins that do not have canonical DNA-related functions. Among the unexpected factors are several metabolic enzymes, oxidative stress response factors, the yeast ubiquitin-activating enzyme, chaperones, proteasome subunits, and RNA-processing factors (S1 Table). Some of these unexpected factors are highly expressed; their binding score may reflect nonspecific binding. On the other hand, not all unexpected interactors are highly expressed or show equal binding scores. In addition, for a substantial number of the identified metabolic enzymes (e.g., glyceraldehyde 3-phosphate dehydrogenase/Tdh3, pyruvate kinase/Cdc19, homocitrate synthase/Lys20,Lys21) and protein chaperones (e.g., the HSP70 chaperones Ssa1 and Ssa2), there is evidence that they have active roles in transcription and DNA repair [54–58]. Furthermore, the binding scores of some metabolic enzymes changed upon treatment with HU (S2 Table), indicating that their interaction with chromatin is not constitutive. Indeed, there is a growing body of evidence that enzymes in the cytoplasm might have moonlighting functions in the nucleus, for example, by local supply of cofactors of histone-modifying enzymes [19,54,59–62]. Alternatively, the interaction with chromatin could somehow influence the activity of metabolic enzymes. The protein interactions that we describe here for 3 barcoded locations provide a rich resource for further unravelling the biological meaning of noncanonical chromatin interactions.

Epi-Decoder is more than a discovery tool. It offers the possibility to compare different loci or to analyse 1 locus under different physiological or genetic conditions in a systematic and quantitative manner. Because all proteins are examined in a pooled fashion using the same affinity handle, differences in purification and cell backgrounds can be excluded and binding scores can be directly compared. This is exemplified by the basal transcription factors Tfa2 and Ssl2, which are quantitative outliers compared to their complex members (TFIIE and TFIIH, respectively), suggesting noncanonical functions. Another example is the chromatin rewiring we observed upon treatment with HU, which uncovers large-scale quantitative changes in interactions of transcription and replication proteins as well as other factors.

We note that Epi-Decoder generates independent binding scores for proteins with (nearly) identical protein sequences but encoded by different genes, such as the histone proteins or other protein paralogs such as Ssa1 and Ssa2, which are 98% identical. This can provide extra information about the differential use of related proteins that cannot, or not easily, be distinguished by MS methods. Protein isoforms that arise through post-translational modification cannot be distinguished by Epi-Decoder.

The Epi-Decoder strategy will be applicable to a broad range of fundamental epigenetic questions. A few requirements need to be met for successful application. First, Epi-Decoder requires a library of tagged proteins. For budding yeast, several tagged protein libraries are already available in addition to the carboxy-terminal TAP-tagged library used here [63–65]. This will allow for extending the current Epi-Decoder analyses to a nearly complete coverage of the proteome as well as to alternative (amino-terminal) tags, together enabling an unprecedented deep analysis of chromatin proteomes. Tagged protein libraries are also becoming available for many other organisms [66–71]. A second requirement for Epi-Decoder applications is the integration of DNA barcodes proximal to the locus of interest. Given their small size, DNA barcodes generally only minimally disrupt a locus, but this needs to be verified for the locus of interest, as is the case for strategies involving targeting affinity handles by Cas9 or other approaches. Fortunately, genomic barcoding has come within reach for many research questions due to the availability of emerging genome engineering strategies, in yeast and other organisms [46,47,72,73]. Indeed, we demonstrate that CRIPSR/Cas9 can be used to generate markerless barcoded DNA libraries at high efficiency for a new locus of interest [46]. Furthermore, our findings with the barcoded native *ADE2* locus demonstrate that Epi-Decoder is not restricted to haploid cells but can also be used in diploid cells in which the tagged allele of a protein and the barcoded allele are present in a heterozygous state. This greatly facilitates creating Epi-Decoder libraries in yeast as well as other cell systems.

In summary, Epi-Decoder is powerful and versatile strategy for decoding the protein interactome of a single genomic locus. We expect that the Epi-Decoder strategy and derivatives thereof will enable the decoding of dynamic proteomes of different loci in different organisms and will be of high value for addressing a broad range of important chromatin biology questions in future applications.

## Methods

### Media and growth conditions

Yeast media was prepared as described previously [12,74]. For screening in untreated conditions, yeast strains were grown in YEPD (1% yeast extract, 2% bacto peptone, and 2% glucose) in log phase. S phase arrest was achieved by adding 1 volume of YEPD plus 360 mM HU to log-phase cells (OD660 of 0.4) in YEPD to achieve 180 mM HU final concentration, and cells were harvested after 2 hours. The arrest was verified by flow cytometry.

### Yeast strains and library manipulations

Yeast strains used in this study are listed in S6 Table. Library manipulations on solid media were performed using SGA technology [12] combined with robotics using a RoToR machine (Singer Instruments, Watchet, UK). Library manipulations in liquid media to generate pools for barcode identification were performed using a Hamilton Microlab Star (Hamilton, Germany). A collection of barcoded TAP-tagged strains (library NKI4217) was generated as follows. In order to cross the TAP-tagged and Barcoder libraries, the TAP-tagged collection [15] was made compatible for SGA and converted to mating type α. This was done by crossing the TAP-tagged library with NKI4212, which was derived from strain Y8205 by replacing the STE2pr-Sp_his5 cassette at the *CAN1* locus by *HphMX* to enable selection during SGA for the TAP-His3MX6 alleles by using histidine prototrophy. This resulted in the *MAT*α TAP-tagged collection (NKI4214) divided over 4 different 1,536-well agar plates. Each of the TAP-tagged plates was mated with the set of 1,140 barcoder *MAT*a strains containing a pair of known unique barcodes flanking a constitutively transcribed kanR marker gene (*KanMX*) under control of the *Ashbya gossypii TEF1* promoter and terminator at the *HO* locus [14]. Diploids were obtained by G418 and Hygromycin double selection on rich media and transferred to sporulation media. After sporulation, the combination library was obtained by selecting twice for *MAT*α haploids and then twice for *MAT*α haploids containing a TAP tag, the barcoded *KanMX* cassette, and the *HphMX* marker. The barcodes and TAP tags were verified for a few selected strains. The effects of sonication time and the presence of methanol in formaldehyde solutions were assessed by performing 3 biological replicate analyses of the pool of plate 2 of the *KanMX* Epi-Decoder library. Five extra barcoded control strains (TAP-tagged versions of Hht2, Htb2, Rpl13a, Ste2, and Bar1) were generated by using the respective clones from the NKI4214 library and introducing unused barcodes from *MAT*a haploid gene knockout library (Open Biosystems, Huntsville, AL) at the *HO* locus of these strains.

### Generation of the BC_5’-ADE2 barcoded collection

#### Preparation of the Cas9/gRNA vector

The Cas9 and gRNA expression vector pML104 (Addgene plasmid number 67638 [75]) was used to introduce a double-strand break at the *ADE2* locus, 57 bp into the coding sequence. pML104 was isolated from dam-INV110 *Escherichia coli* (ThermoFisher number C717103) and digested with SwaI (NEB number R0604) and BclI (NEB number R0160). DNA oligonucleotides gRNA_Ade2_39a and gRNA_Ade2_39b (designed using http://wyrickbioinfo2.smb.wsu.edu/crispr.html) were hybridized in T4 ligation buffer (50 mM Tris-HCl, 10 mM MgCl_2_, 1 mM ATP, 10 mM DTT [pH 7.5]) cycling through the following: 95 °C for 6 minutes; decrease 70× 1 °C/1 minute (final 25 °C). The hybridized oligonucleotides were directly ligated into the digested pML104 vector (molar ratio: 100 hybridized nucleotide insert per 1 cut vector) by T4 ligase (NEB number M0202S) for 20 hours at 16 °C. Alpha-Select Silver Competent Cells (Bioline number BIO-85026) were transformed by heat shock with the ligation product for amplification of the ADE2-gRNA–containing pML104 vector (pIla019).

#### Barcode-containing repair template

The barcode-containing repair template was generated from a long DNA oligonucleotide (Repair_Guide_39; final concentration 0.001 μM) using MyTaq Red Mix (Bioline number BIO-25043) and 2 primers (Ade2_BC_39_fw and Ade2_BC_39_rv; final concentration 0.5 μM) in a PCR reaction: 96 °C 1 minute, 14× (96 °C 20 seconds, 50 °C 20 seconds, 72 °C 20 seconds) and 72 °C 1 minute. The PCR product was purified using CleanPCR beads (CleanNA number CPCR-0050).

#### Preparation of *S*. *cerevisiae* with a selection marker

Before introducing a barcode, the selection marker *NatMX* was introduced 140 bp downstream of the *ADE2* ORF to enable selection for the barcoded locus during genetic crosses with the TAP-tagged protein collection. This step can be omitted for screens with diploid cells. *NatMX* was amplified from pFvL99 [76] (primers: pRS_to_ADE2_1 and pRS_to_ADE2_2) and integrated in BY4741 [77] by homologous recombination followed by CloNat (100 μg/mL) resistance selection, resulting in strain NKI2558.

#### Construction of the BC_5’-ADE2 collection

NKI2558 (4 × 10^6^ cells) was transformed with the ADE2-gRNA–containing vector pIla019 (50–250 ng) and repair template (12.5–125 pM) via the LiAc/SS carrier DNA/PEG method [78]. Transformed cells were plated on synthetic complete uracil drop-out plates and colonies were grown for 4 days at 30 °C. Single colonies were moved to liquid YEPD plus CloNat in 384-well plates using toothpicks. These arrayed strains were plated using the RoToR from Singer Instruments (Watchet, UK) on solid medium containing 5-fluoroorotic acid to select for the loss of the plasmid. The new BC_5’-ADE2 collection (NKI2559) was crossed with the TAP-tagged collection as described in Methods section ‘Yeast strains and library manipulations’, with the exception that CloNat was used instead of G418 to select for the barcoded allele in this library. The diploid library (NKI2560) was used for Epi-Decoder analysis.

#### Decoding the barcode sequences and locations of the BC_5’-ADE2 collection

The BC_5’-ADE2 collection was grown in 384-well plates in liquid YEPD plus CloNat, and a Hamilton robotics liquid handling workstation (Hamilton Microlab Star, Hamilton, Germany) was used to make pools of strains that were located in the same row or in the same column so that yeast from each position on the plate was present in 1 row pool and 1 column pool [79]. DNA was extracted from each pool using phenol-chloroform-isoamyl alcohol (Methods section: ‘Genomic DNA isolation and copy number determination by sequencing’). Barcodes from all the pools were amplified by PCR to prepare sequencing libraries for the MiSeq platform, similar to what was described in Methods section ‘Epi-Decoder (TAG-ChIP-Barcode-Seq)’. During this barcode amplification, a pool-specific unique 6 or 12 bp index was incorporated in the PCR products (S7 Table). High-throughput sequencing was performed using a custom sequence primer following the guidelines from http://nextgen.mgh.harvard.edu/CustomPrimer.html. After high-throughput sequencing, reads were sorted by the pool-specific index. The first 15 bp sequence-reads that occurred at least 50 or 100 times with the same index were appointed to be the barcodes. Barcodes identified in a single-row pool were searched for in all single-column pools; a match between row and column reflects a coordinate, a position on the original 384-well plate. Barcodes for which more than one matching row–column combination was detected were rejected. Locations to which more than one barcode was assigned were also rejected.

### Epi-Decoder (TAG-ChIP-Barcode-Seq)

Epi-Decoder libraries were grown on YEPD plates overnight, and the colonies of each plate were pooled together in liquid culture. The cultures were grown until log phase (OD660 of approximately 0.4) and crosslinked for 20 minutes with one-tenth of the volume of freshly prepared Fix Solution (1% formaldehyde [Sigma-Aldrich 252549-500ML], 50 mM Hepes-KOH [pH 7.5], 100 mM NaCl, 1 mM EDTA) and subsequently quenched for 5 minutes with Glycine (125 mM final concentration). Where indicated, methanol-free formaldehyde (Thermo Scientific; 28908) was used for crosslinking. Cells were washed once in cold TBS with 0.2 mM PMSF, and the pellet was frozen at −80 °C. Cells from frozen pellets of approximately 1.5 × 10^9^ cells were lysed by bead beating in 200 μL breaking buffer (100 mM Tris [pH 7.9], 20% glycerol, protease inhibitor cocktail EDTA-free) with Zirconia/silica beads. The lysate was washed twice in 1 mL FA buffer (50 mM HEPES-KOH [pH 7.5], 140 mM NaCl, 1 mM EDTA, 1% Triton X-100, 0.1% Na-deoxycholate, protease inhibitor cocktail EDTA-free) and sonicated using the Bioruptor PICO (Diagenode) for 10 minutes at 30-second intervals. Where indicated, chromatin was sonicated for 5 or 20 minutes. Chromatin was cleared by centrifugation for 5 minutes at 4 °C at 4,000 rpm. The amount of 100 μL chromatin was used as input material. For ChIP of the Barcoded KanMX gene, immunoglobulin G (IgG) Sepharose 6 Fast Flow beads (GE Healthcare) were washed 3 times with PBSB (PBS containing 5 mg/mL BSA) and incubated with 1 mL chromatin for 6 hours on a turning wheel at 4 °C. For ChIP of the barcoded *ADE2* locus, Dynabeads M-270 Epoxy beads (ThermoFisher number 14301) were used. Rabbit IgG (0.2 mg) from serum (Sigma, I5006-100MG) was coupled to 10 mg epoxy-activated Dynabeads in phosphate buffer with ammonium sulphate according to the manufacturer’s protocol. After a PBS wash, IgG-coupled beads were resuspended in 1.2 mL PBS containing 0.02% sodium azide. Of a prepared beads solution, 80 μL was used for 800 μL chromatin. Samples were washed twice in FA buffer, twice in high-salt FA buffer (500 mM NaCl), twice in RIPA buffer (10 mM Tris [pH 8], 250 mM LiCl, 0.5% NP-40, 0.5% Na-deoxycholate, 1 mM EDTA) and once with TE buffer (10 mM Tris [pH 8], 1 mM EDTA). With each wash step, the Sepharose beads were spun for 2 minutes at 3,000 rpm at 4 °C. IP samples were eluted for 10 minutes at 65 °C in 100 μL elution buffer (50 mM Tris [pH 8], 10 mM EDTA, 1% SDS). IP and input samples were digested with 0.5 μL RNase A (10 mg/mL) and 10 μL ProtK (10 mg/mL) in 70 μL TE for 1 hour at 50 °C and subsequently kept overnight at 65 °C to reverse crosslinks. DNA was purified using the QIAquick PCR purification kit (Qiagen). BC_UP, BC_DN and BC_5’_ADE2 were amplified separately with specific primers (S7 Table: 2B_UP_Fw + 2B_UP_Rv_all; 2B_DN_Fw +2B_DN_Rv_all; P5-XL-ADE2_g39_REV + P7L-IDn-ADE2_g39_REV). PCR products were mixed in an equimolar fashion and purified from an agarose gel with a size selection of 100 to 150 bp. The purified DNA was sequenced (single read, >50 bp) on a HiSeq2500/MiSeq platform (Illumina, San Diego, CA), using one or a mix of custom sequencing primers (S7 Table: 2B_UP_Seq, 2B_DN_Seq or Seq-XL-ADE2_g39_REV).

### Barcode counting

Barcodes were extracted from the sequencing reads by using the Perl script eXtracting Counting and LInking to Barcode References (XCALIBR). The code and detailed descriptions of the functions are available at https://github.com/NKI-GCF/xcalibr. Briefly, XCALIBR locates the constant regions (U2 and D2 of the barcoded *KanMX* gene or the constant region next to the barcode at *ADE2*) in each amplicon and reports the 6 bp upstream of the index and 20 bp downstream of the KanMX barcode or 15 bp downstream of the *ADE2* barcode. The resulting table contains counts for each barcode–index combination. Counts below 10 were removed from this table before further preprocessing and filtering steps.

### Barcode data preprocessing and filtering

Input (IN) and immunoprecipitated DNA (IP) of each plate were amplified separately with a unique index. Even though we aimed to mix each sample equimolarly, plate-specific differences in counts could still occur. This was corrected by normalizing each plate by its median. The counts table was log2 transformed, and barcode-index combinations were matched with the ORF names. Factors with low input counts were removed because these were likely to be missing from the library or the barcode failed to amplify due to technical reasons. We manually validated several factors for the presence of a TAP tag by PCR with primers in the specific ORF and the TAP tag. This revealed that ORC4, MCM3, MCM7, and RAD6 were not properly tagged; we therefore removed these strains for further analysis. The final set of the barcoded KanMX library contained information on 3,994 BC_UP and 3,955 BC_DN barcode clones. We noticed that the dynamic range was slightly different between biological replicates. To overcome this problem, we performed quantile normalisation for the replicates of BC_UP and BC_DN separately. This method is used to generate similar distributions by first ranking each replicate based on the counts and then replacing the counts of each rank by the mean count of that rank.

### Barcode statistical analysis

Factors with IP counts that were significantly enriched over input were identified by using the Limma R/Bioconductor software package [80]. *p*-Values were adjusted for multiple testing by converting them to FDRs using the Benjamini-Hochberg procedure. Factors with a positive fold-change and an FDR < 0.01 were selected as significantly enriched. For untreated conditions, 6 independent biological samples were used. The running sum scores for the enrichment plot (Fig 3B) were calculated with the gseaScores function from the HTSanalyzeR package. For identifying differential binders upon HU treatment, 3 biological samples were used for treatment and no treatment. Here, we only considered factors that were significant binders in at least one of the conditions (FC > 0 and FDR < 0.05). Limma was used to select factors with significantly different IP counts (FDR < 0.05). Barcode counts were compared with protein abundance data measured by GFP intensity. This data was obtained from the CYCLoPs database [81].

### GO slim process enrichment

GO slim process terms are condensed versions of the full GO ontology [82,83]. GO slim process terms were downloaded from the Saccharomyces Genome Database [84], and enrichment analysis was performed by using the fisher.test function in R with option alternative ‘greater’. We manually assigned the following categories based on GO slim terms listed here: DNA binding: DNA-templated transcription, initiation, DNA-templated transcription, elongation, DNA-templated transcription, termination, transcription from RNA polymerase I promoter, transcription from RNA, polymerase II promoter, chromatin organisation, histone modification, DNA replication, DNA recombination, DNA repair, cellular response to DNA damage stimulus, regulation of DNA metabolic process, nuclear transport, and chromosome segregation; RNA binding or processing: mRNA processing, rRNA processing, tRNA processing, RNA modification, RNA splicing, and RNA catabolic process; and metabolism: carbohydrate metabolic process, cofactor metabolic process, nucleobase-containing small-molecule metabolic process, monocarboxylic acid metabolic process, cellular amino acid metabolic process, generation of precursor metabolites and energy, oligosaccharide metabolic process, and lipid metabolic process.

### ChIP-qPCR

ChIP experiments were performed similar to TAG-ChIP-Barcode-Seq but with 20 μl bed volume of IgG Sepharose 6 Fast Flow beads and 200 μl chromatin. For untagged-H2A.Z ChIP, the Htz1-specific antibody (Active Motif, 39647) was coupled to Protein G Dynabeads (Thermo Fisher Scientific). ChIP was performed with 20 μl Dynabeads and 200 μl chromatin. Factors were selected such that they reflect different classes of binders and different expression levels, in addition to negative controls Bar1 (not expressed in these cells) and Rpl13A (a ribosomal subunit). qPCR was performed on the purified DNA with SYBR green master mix (Applied Biosystems or Roche) or SensiFAST SYBR master mix (Bioline) according to the manufacturer’s protocol and analysed on LightCycler 480 II (Roche). The binding was analysed with specific primers in close proximity to the BC_UP and BC_DN (S7 Table). Each sample was measured in 2 technical duplicates in the qPCR, and the average value of these 2 was taken as 1 value when combining biological replicates. For comparison of the ChIP-qPCR and BC-seq results in a quantitative manner, the negative control (Bar1) was used to normalize the raw values.

### Flow cytometry

Flow cytometry samples were prepared to monitor cell cycle progression and verify S phase arrest by HU. A total of 1 × 10^7^ cells were collected and fixed with 70% ethanol and stored at −20 °C. Flow cytometry was performed as previously described [76] after staining DNA with Sytox green (Molecular Probes). Flow cytometry measurements were taken on a FACSCalibur with CellQuest software (Becton Dickinson) and further analysed with FlowJo software (Treestar).

### Genomic DNA isolation and copy number determination by sequencing

Two independent strains (NKI8529 and NKI8587) were selected for genomic DNA isolation to determine the replication status of genomic loci. Genomic DNA was isolated as described previously [85]. Briefly, 2 × 10^7^ cells were spun down and resuspended in 200 μl of 2% Triton X-100, 1% SDS, 100 mM NaCl, 10 mM Tris [pH 8.0], and 1 mM EDTA. Then, 200 μl phenol-chloroform-isoamyl alcohol (25:24:1) was added, and cells were lysed by bead beating with 300 μl Zirconia/silica beads. TE 200 μl was added, the cells were spun for 5 minutes at maximum speed, and the aqueous layer was transferred to a new tube. The pellet was washed in 1 mL 100% ethanol and resuspended in 400 μl TE. To precipitate the DNA 10, approximately 14 M ammonium acetate and 1 mL 100% ethanol were added, and the tube was inverted to mix contents and spun 2 minutes at 13,000 rpm. The pellet was air dried and suspended in 50 μl TE. The gDNA was then purified by sending through an Isolate II genomic DNA kit (Bioline). The amount of 20 μl of ethanol-precipitated gDNA was resuspended in 180 μl lysis buffer GL and 1 μl RNaseA (10 mg/mL) and kept at room temperature for 20 minutes before proceeding with step 3 of the manufacturer’s protocol. DNA was eluted in 50 μl elution buffer G. The gDNA was sheared with a Covaris E220 ultrasonicator (Covaris) to obtain fragments of 150 to 200 bp and sequenced on the HiSeq2000 platform (Illumina, San Diego, CA).

### Genomic DNA isolation and sequencing data analysis

Single-end reads of 65 bp were aligned to the *S*. *cerevisiae* reference genome version R64 (UCSC SacCer3) by using BWA [86]. The BAM files with aligned reads were filtered for mapq 37 and converted to bedgraph with bins of 250 bp by using deepTools [87]. By using custom R scripts, the region of unstable transcript XUT_12F-188 was removed because it contains repetitive sequences. The average coverage in each 250 bp bin was plotted across the entire genome by using custom R scripts. The replication timing in the absence of HU was obtained from a density transfer experiment [36] and downloaded from S1 Table in that study. This table contains the percentage of the genome that had become hydrid in density (percent HL DNA) in cells collected at different time points [36]. The plots were generated for both samples separately and showed similar patterns.

### Reverse transcription

RNA was isolated using the RNeasy Mini Kit (QIAGEN) using the protocol for yeast cells, with a few modifications. Briefly, 2 × 10^7^ cells were spun down (5 minutes at 3,000 rpm), and pellets were dissolved in 600 μL cold RLT buffer. Cells were broken by bead beating with 400 μL Zirconia/silica beads, and debris was separated by centrifuging 2 minutes at 13,000 rpm. The supernatant (approximately 350 μL) was collected and mixed with one volume (approximately 350 μL) 70% EtOH and transported to RNeasy columns. Following the buffer RW1 and buffer RPE wash steps, RNA was eluted in 50 uL elution buffer. Eluted RNA was treated with DNase I (QIAGEN) to remove genomic DNA. Next, cDNA was prepared using SuperScript II reverse transcriptase (Invitrogen). Reverse transcription PCR (RT-PCR) was performed with the primers in S7 Table. Each sample was measured in 2 technical duplicates, and the average value of these 2 was taken as 1 value when combining biological replicates.

### Quantitative data used for graphs in figures

Quantitative observations that underlie the data summarised in the graphs are shown in S1 Data.

## Supporting information

S1 Fig Relates to Fig 1. Schematic overview of the primers used to amplify the HO barcodes, which are flanked by constant regions U1, U2, D1, and D2. The forward primers introduce the Illumina P5 sequence and extra nucleotides for annealing of the 5’ end of the custom sequencing primers. The reverse primers introduce the Illumina P7 sequence as well as a 6 bp index.
(TIF)

S2 Fig Relates to Fig 2. (A) Scatter plot showing the normalized BC-UP and BC-DN barcode counts for the binders of biological replicates. Two samples were randomly chosen for a representative figure. The mean correlation and SD was calculated for all 6 replicates. (B) Bar plot showing the GO slim terms (process) enriched at BC_UP and BC_DN. Terms with a Fisher Exact *p*-value <0.01 were considered to be enriched. The x-axis shows the number of factors associated with each term, and the nontransparent colour indicates the proportion of binders. (C) ChIP-qPCR analysis of selected TAP-tagged strains with specific primers in proximity to BC_UP and BC_DN (S7 Table). The average of 3 biological replicates is shown; the error bars indicate the SD. Bar1 (not expressed) and Rpl13a (a highly expressed ribosomal subunit) were included as negative controls. Rpo21 (the largest subunit of RNA polymerase II), Orc1 (the largest subunit of ORC), and Lys20 (homocitrate synthase isozyme) were identified as binders by Epi-Decoder. Ilv2 (acetolactate synthase), Rnr1 (a subunit of ribonucleotide reductase), Pfk1 (phosphofructokinase), and Eft1 (a translation elongation factor) are highly expressed proteins (see panel D) that were not identified as binders by Epi-Decoder. (D) Expression levels of proteins shown in panel C were verified by immunoblot analysis. The indicated protein sizes (kDa) include the TAP tag. Untagged histone H3 was used as a loading control; the asterisks indicate nonspecific bands that are also detected in the negative Bar1 control. (E) Scatter plot showing the normalized barcode counts for one subset of the HO Barcoder library (see Methods and S3 Table), comparing regular methanol-containing with methanol-free formaldehyde. Values shown are average across 3 replicates for both BC_UP and BC_DN. Underlying data for S2B and S2C Fig in S1 Data.
(TIF)

S3 Fig Relates to Fig 3. (A) ChIP-qPCR analysis of endogenous (untagged) H2A.Z with specific primers in proximity to BC_UP and BC_DN (S7 Table). Two independent biological replicates are shown of strain NKI3504. (B) DNA gel showing the distribution of the DNA fragment sizes after 5-, 10-, and 20-minute sonication, with average values of 520, 470, and 390 bp. (C) Bar plot showing normalized Epi-Decoder barcode counts in the 5-, 10-, and 20-minute sonication samples. Shown are factors of the TFIIH complex that were specifically enriched at BC_UP (promoter) and subunits of ORC and MCM that were specifically enriched at BC_DN (proximal to the origin of replication). Bars represent average across 3 biological replicates, and error bars represent SD. Underlying data for S3A and S3C Fig in S1 Data.
(TIF)

S4 Fig Relates to Fig 4. (A) Flow cytometry analysis showing the DNA content of cells with (“HU”) and without (“UN”) hydroxyurea treatment (180 mM for 2 hours). (B) Volcano plot similar to Fig 4b, but here, the dots are coloured based on their protein abundance changes in HU based on data from [88]. (C) ChIP-qPCR for selected strains, with specific primers in close proximity to BC_UP and BC_DN. Bar1-TAP was used as a negative control because it is not expressed in these cells. To compare the barcode counts with ChIP-qPCR signal, the samples were normalized by the Bar1-TAP signal before calculating ChIP/input. The average of 3 biological replicates is shown; the error bars indicate SD. Underlying data for S4C Fig in S1 Data.
(TIF)

S5 Fig Relates to Fig 5. Design and expression of the barcoded *ADE2* locus. (A) *NatMX6* (pink) was inserted downstream of the *ADE2* (YOR128C) coding sequence (orange), after which 15 bp barcodes were inserted 57 bp downstream of the start codon of *ADE2* by template-directed repair of a CRISPR/Cas9-induced break. The zoom-in visualizes the repair template that was transformed in to *S*. *cerevisiae* along with the Cas9 plus gRNA expression vector by which the barcode was inserted: the orange bar is the 5’ end of *ADE2*; 15× ‘N’ is the barcode; the yellow bar (minus the 15× ‘N’) is complementary to gRNA plus the PAM sequence (red bar); the grey bars are the homology arms. The purple delineated arrows are primers used for the following: amplifying the repair template (black text, Ade2_BC_39_fw and Ade2_BC_39_rv); amplifying an Epi-Decoder_5’_ADE2 library (red text, P5-XL-ADE2_g39_REV and P7L-ID7_ADE2_g39_REV); sequencing an Epi-Decoder BC_5’-ADE2 library (green text, Seq-XL-ADE2_g39_REV). (B) The relative activity of the *ADE2* and *AgTEF1* promoter was derived from transcript levels of the mRNAs expressed from the 2 promoters (*ADE2* and *Hph*) as determined by RT-qPCR. qPCR was also performed for gDNA samples (*n* = 9) to correct for differences in primer efficiency. The bars represent 2 biological replicates (strains NKI8587 and NKI8588; arbitrary values). *ADE2* mRNA (expressed by the *ADE2* promoter) was lower than Hph mRNA (expressed by the *AgTEF1* promoter of the HphMX cassette). The lower expression of *ADE2* is in agreement with RNA-Seq measurements of relative *ADE2* and *ScTEF1* expression levels [50]. Underlying data for S5B Fig in S1 Data.
(TIF)

S1 Table List of strains in the Epi-Decoder library and their associated binding scores. For each TAP-tagged strain, the corresponding barcode enrichment (average log2 fold-change [logFC] of ChIP/In) and Benjamini-Hochberg-corrected *p*-value (FDR) are provided for both BC_UP and BC_DN. The GO_cat column contains the general categories based on GO slim process terms. The complex column contains several well-known protein complexes that bind DNA.
(XLSX)

S2 Table List of binders in either untreated or HU-treated cells and their associated binding difference. This table contains the significant binders in either untreated or HU treatment. For each strain, the average log2 fold-change (logFC) of HU-treated/untreated and their associated Benjamini-Hochberg-corrected *p*-value (FDR) are provided for both BC_UP and BC_DN. The complex column contains several well-known protein complexes that bind DNA.
(XLSX)

S3 Table ChIP/input barcode counts for samples fixated by using formaldehyde with or without methanol. This table includes experiments of 3 biological replicates using methanol-free (UP_met_free, DN_met_free) and methanol-containing formaldehyde (UP_met, DN_met).
(XLSX)

S4 Table ChIP/input barcode counts for samples sonicated for 5, 10, or 20 minutes. This table includes both BC_UP and BC_DN and experiments of 3 biological triplicates, R1, R2, and R3.
(XLSX)

S5 Table ChIP/input barcode counts for BC_5’-ADE2 at the *ADE2* locus. This table includes data of 2 biological replicates, rep1 and rep2.
(XLSX)

S6 Table List of yeast strains used in this study. This file contains the yeast strains used in this study.
(XLSX)

S7 Table List of the DNA oligonucleotides used in this study. This file contains the DNA oligonucleotides used in this study.
(XLSX)

S1 Data This file contains the quantitative observations that underlie the data summarized in the graphs included in the manuscript. (XLSX)

## Abbreviations

BC_5’-ADE2: barcode in 5’ region of *ADE2*
BC_DN: downstream barcode at HO locus
BC_UP: upstream barcode at HO locus
ChIP: chromatin immunoprecipitation
CRISPR/Cas9: clustered regularly interspaced short palindromic repeats and CRISPR-associated protein 9
dNTP: deoxyribonucleotide triphosphate
FDR: false discovery rate
GFP: green fluorescent protein
GO: gene ontology
gRNA: guide RNA
MCM: minichromosome maintenance
MS: mass spectrometry
ORC: origin recognition complex
qPCR: quantitative PCR
RT-PCR: reverse transcription PCR
SGA: synthetic genetic array
TAP: Tandem Affinity Purification
Top2: topoisomerase II
