# Supplementary figures and images for: Decoding the chromatin proteome of a single genomic locus by DNA sequencing
Source: PLoS Biol. 2018 Jul 13;16(7):e2005542. doi: 10.1371/journal.pbio.2005542 (PMC6059479; doi:10.1371/journal.pbio.2005542)

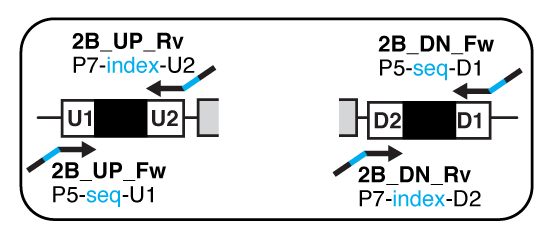

Supplement: S1 Fig — Schematic overview of the primers used to amplify the HO barcodes, which are flanked by constant regions U1, U2, D1, and D2. The forward primers introduce the Illumina P5 sequence and extra nucleotides for annealing of the 5’ end of the custom sequencing primers. The reverse primers introduce the Illumina P7 sequence as well as a 6 bp index. (TIF) [file pbio.2005542.s001.tif]

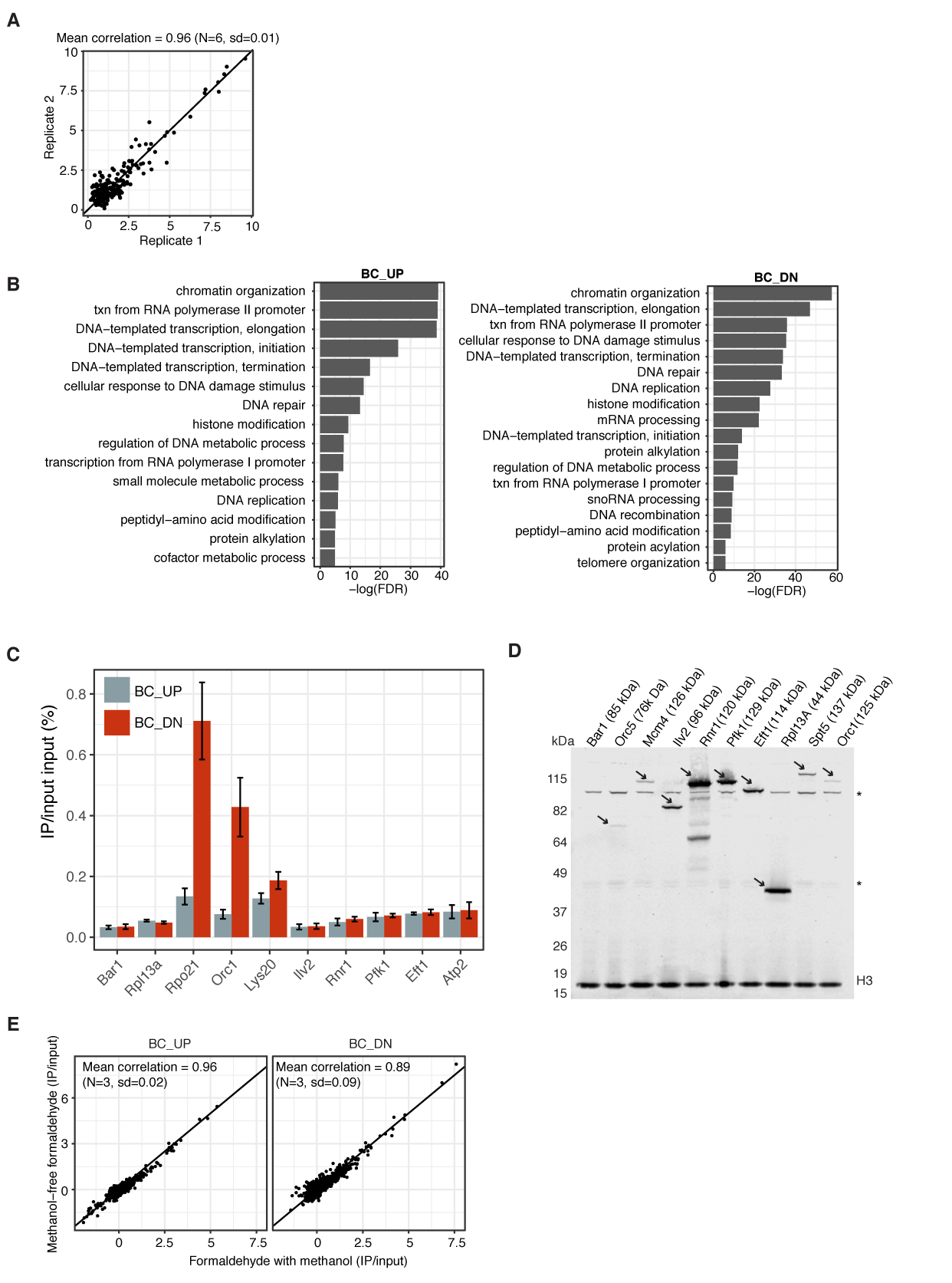

Supplement: S2 Fig — (A) Scatter plot showing the normalized BC-UP and BC-DN barcode counts for the binders of biological replicates. Two samples were randomly chosen for a representative figure. The mean correlation and SD was calculated for all 6 replicates. (B) Bar plot showing the GO slim terms (process) enriched at BC_UP and BC_DN. Terms with a Fisher Exact p-value <0.01 were considered to be enriched. The x-axis shows the number of factors associated with each term, and the nontransparent colour indicates the proportion of binders. (C) ChIP-qPCR analysis of selected TAP-tagged strains with specific primers in proximity to BC_UP and BC_DN (S7 Table). The average of 3 biological replicates is shown; the error bars indicate the SD. Bar1 (not expressed) and Rpl13a (a highly expressed ribosomal subunit) were included as negative controls. Rpo21 (the largest subunit of RNA polymerase II), Orc1 (the largest subunit of ORC), and Lys20 (homocitrate synthase isozyme) were identified as binders by Epi-Decoder. Ilv2 (acetolactate synthase), Rnr1 (a subunit of ribonucleotide reductase), Pfk1 (phosphofructokinase), and Eft1 (a translation elongation factor) are highly expressed proteins (see panel D) that were not identified as binders by Epi-Decoder. (D) Expression levels of proteins shown in panel C were verified by immunoblot analysis. The indicated protein sizes (kDa) include the TAP tag. Untagged histone H3 was used as a loading control; the asterisks indicate nonspecific bands that are also detected in the negative Bar1 control. (E) Scatter plot showing the normalized barcode counts for one subset of the HO Barcoder library (see Methods and S3 Table), comparing regular methanol-containing with methanol-free formaldehyde. Values shown are average across 3 replicates for both BC_UP and BC_DN. Underlying data for S2B and S2C Fig in S1 Data. (TIF) [file pbio.2005542.s002.tif]

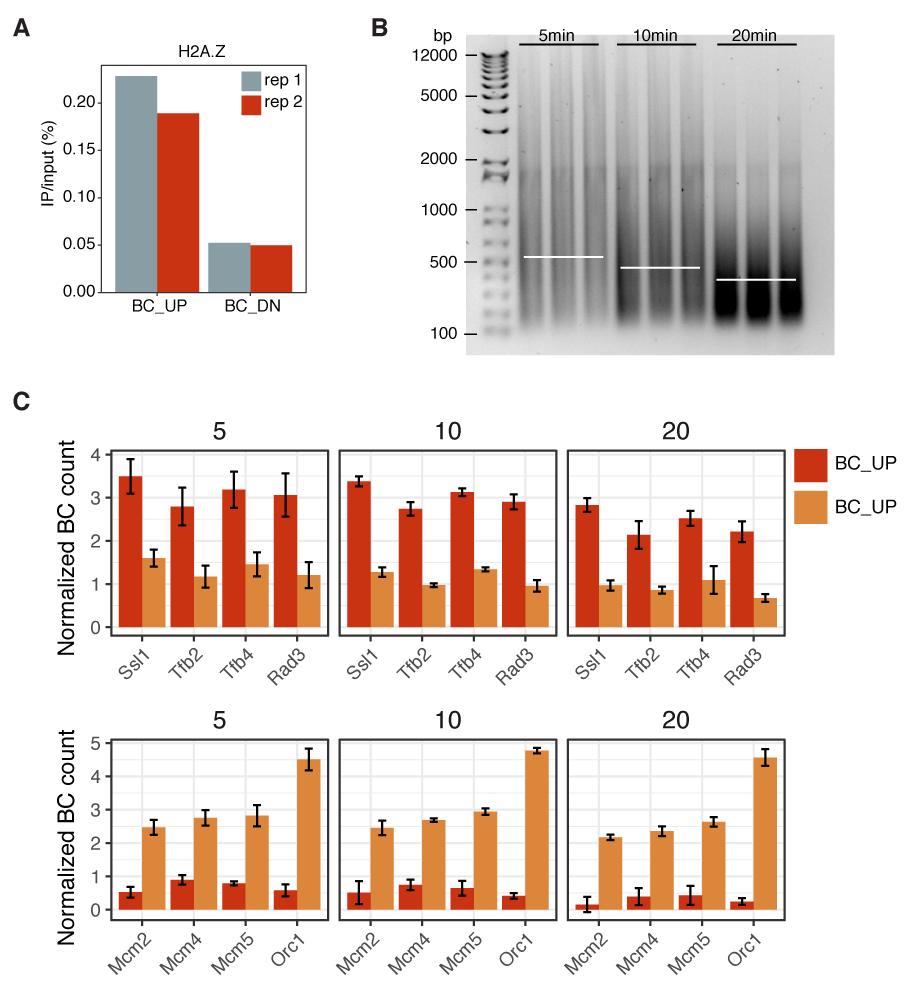

Supplement: S3 Fig — (A) ChIP-qPCR analysis of endogenous (untagged) H2A.Z with specific primers in proximity to BC_UP and BC_DN (S7 Table). Two independent biological replicates are shown of strain NKI3504. (B) DNA gel showing the distribution of the DNA fragment sizes after 5-, 10-, and 20-minute sonication, with average values of 520, 470, and 390 bp. (C) Bar plot showing normalized Epi-Decoder barcode counts in the 5-, 10-, and 20-minute sonication samples. Shown are factors of the TFIIH complex that were specifically enriched at BC_UP (promoter) and subunits of ORC and MCM that were specifically enriched at BC_DN (proximal to the origin of replication). Bars represent average across 3 biological replicates, and error bars represent SD. Underlying data for S3A and S3C Fig in S1 Data. (TIF) [file pbio.2005542.s003.tif]

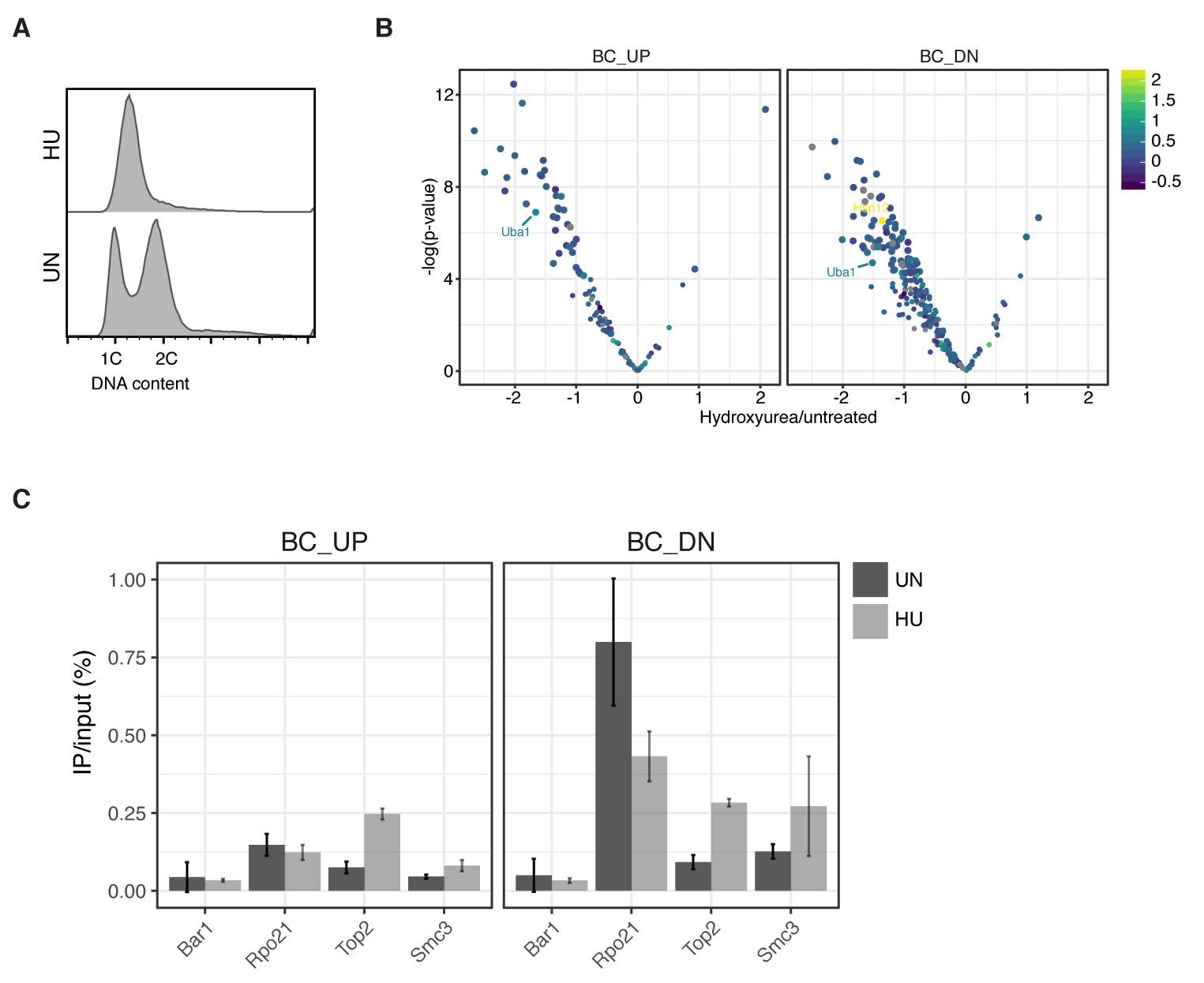

Supplement: S4 Fig — (A) Flow cytometry analysis showing the DNA content of cells with (“HU”) and without (“UN”) hydroxyurea treatment (180 mM for 2 hours). (B) Volcano plot similar to Fig 4b, but here, the dots are coloured based on their protein abundance changes in HU based on data from [88]. (C) ChIP-qPCR for selected strains, with specific primers in close proximity to BC_UP and BC_DN. Bar1-TAP was used as a negative control because it is not expressed in these cells. To compare the barcode counts with ChIP-qPCR signal, the samples were normalized by the Bar1-TAP signal before calculating ChIP/input. The average of 3 biological replicates is shown; the error bars indicate SD. Underlying data for S4C Fig in S1 Data. (TIF) [file pbio.2005542.s004.tif]

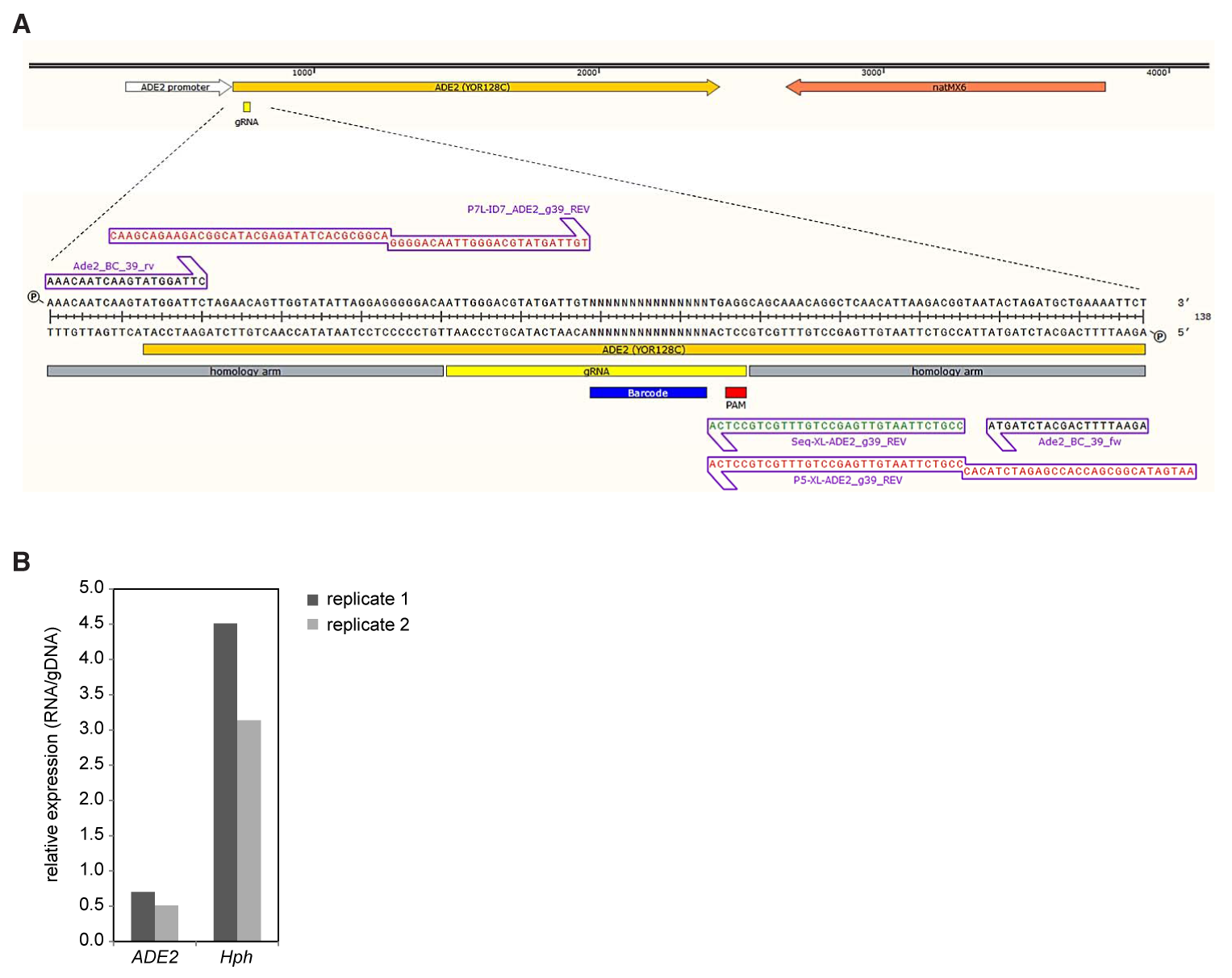

Supplement: S5 Fig — Design and expression of the barcoded ADE2 locus. (A) NatMX6 (pink) was inserted downstream of the ADE2 (YOR128C) coding sequence (orange), after which 15 bp barcodes were inserted 57 bp downstream of the start codon of ADE2 by template-directed repair of a CRISPR/Cas9-induced break. The zoom-in visualizes the repair template that was transformed in to S. cerevisiae along with the Cas9 plus gRNA expression vector by which the barcode was inserted: the orange bar is the 5’ end of ADE2; 15× ‘N’ is the barcode; the yellow bar (minus the 15× ‘N’) is complementary to gRNA plus the PAM sequence (red bar); the grey bars are the homology arms. The purple delineated arrows are primers used for the following: amplifying the repair template (black text, Ade2_BC_39_fw and Ade2_BC_39_rv); amplifying an Epi-Decoder_5’_ADE2 library (red text, P5-XL-ADE2_g39_REV and P7L-ID7_ADE2_g39_REV); sequencing an Epi-Decoder BC_5’-ADE2 library (green text, Seq-XL-ADE2_g39_REV). (B) The relative activity of the ADE2 and AgTEF1 promoter was derived from transcript levels of the mRNAs expressed from the 2 promoters (ADE2 and Hph) as determined by RT-qPCR. qPCR was also performed for gDNA samples (n = 9) to correct for differences in primer efficiency. The bars represent 2 biological replicates (strains NKI8587 and NKI8588; arbitrary values). ADE2 mRNA (expressed by the ADE2 promoter) was lower than Hph mRNA (expressed by the AgTEF1 promoter of the HphMX cassette). The lower expression of ADE2 is in agreement with RNA-Seq measurements of relative ADE2 and ScTEF1 expression levels [50]. Underlying data for S5B Fig in S1 Data. (TIF) [file pbio.2005542.s005.tif]
